# Rab1A promotes cell proliferation and migration by upregulating Gli1 in colorectal cancer

**DOI:** 10.1038/s41598-021-95798-7

**Published:** 2021-08-10

**Authors:** Chaozhong Peng, Xiao Li, Zhixue Ye, Wenqing Wu

**Affiliations:** Department of General Surgery, Suzhou Wuzhong People’s Hospital, No. 61 Dongwu North Road, Suzhou, 215128 Jiangsu China

**Keywords:** Cancer, Gastrointestinal cancer, Oncogenes

## Abstract

Rab1A, as a highly conserved small guanosine triphosphatase (GTPase), plays contentious roles in different types of cancers. The role of Rab1A in colorectal cancer (CRC) has been described in previous studies, but the molecular mechanisms of Rab1A in CRC remain far from being addressed. In the present study, we found that Rab1A expression was significantly upregulated in CRC tissues and increased Rab1A expression correlated with tumor size, lymph node metastasis (LNM) and tumor-node-metastasis (TNM) stage of CRC patients. We also found that Rab1A exerts its promotive effect on CRC cell proliferation, migration and EMT progress. Further mechanistic experiments showed that glioma-associated oncogene-1 (Gli1), as a key transcriptional factor of the Hedgehog pathway, was implicated in Rab1A-mediated regulation of CRC cell proliferation and migration. In addition, Rab1A upregulated Gli1 expression through Smoothened homolog (SMO)-independent pathway. Finally, Rab1A activated mechanistic target of rapamycin (mTOR) signaling in CRC cells. Collectively, our results define Rab1A as a novel regulator of Gli1 to promote CRC cell proliferation and migration, and suggest that the Rab1A/mTOR/Gli1 axis may serve as a promising therapeutic target for the treatment of CRC.

## Introduction

Colorectal cancer (CRC) is an important digestive tract tumor with high malignancy and is the second leading cause of cancer-related deaths globally^1,2^. Although there have been significant improvements in treatment of CRC, the prognosis of advanced and metastatic CRC patients is poor because of lack of effective targets for treatment^3^. Various studies have explored the genetic and epigenetic biomarkers for CRC risk^4–6^, but the underlying pathogenesis of CRC is not yet completely elucidated. Therefore, more effective biomarkers and therapeutic targets are needed to improve the diagnosis and treatment of CRC.

Rab1A, as a highly conserved small guanosine triphosphatase (GTPase), predominantly regulates intracellular vesicular trafficking from the endoplasmic reticulum (ER) to the Golgi apparatus^7,8^. It has been reported that Rab1A protein is involved in mediating signal transduction^9^ and cell migration^10^. Recently, Rab1A has been described as an oncogene in multiple types of cancers, such as gastric cancer^8^, nasopharyngeal carcinoma^11^, breast cancer^12^, lung cancer^13^ and hepatocellular carcinoma^14^. On the other hand, it was reported that Rab1A expression was reduced in androgen-independent prostate cancer and Rab1A depletion enhanced the proliferation ability of prostate cancer cells^15^. Previous researches have shown that Rab1A is a colorectal oncogene and can promote CRC cell proliferation and migration via regulating the mTOR/S6K1 pathway^16,17^. However, the precise functional roles and the underlying molecular mechanism of Rab1A in CRC remain far from being addressed.

The Hedgehog (HH) signaling pathway is a conserved pathway involved in cell growth and tissue patterning^18–20^. In mammals, HH signaling initiates with binding of one of the three ligands (Indian Hedgehog (IHH), Sonic Hedgehog (SHH) and Desert Hedgehog (DHH)) to the protein patched homolog (PTCH), causing the release of the suppressed transmembrane protein Smoothened (SMO). The release of SMO subsequently activates the Gli1 transcription factors^21^. The SMO-dependent Gli1 activation is referred to as the canonical HH signaling^22^. Alternatively, Gli1 can be activated independently of PTCH or SMO, which is then termed as non-canonical HH signaling^22,23^. The signaling pathways that can induce non-canonical Gli1 activation includes extracellular signal regulated kinase (ERK) signaling and phosphoinositide 3-kinase (P13K)/mammalian target of rapamycin (mTOR) signaling pathway^24–26^.

It was previously reported that Rab1A expression positively correlated with Gli1 in gastric cancer tissues^27^, but whether Gli1 is regulated by Rab1A in CRC cells remains unknown. In the present study, we found that Rab1A was highly expressed and exerted oncogenic roles in CRC. Further mechanistic experiments revealed that Rab1A promoted CRC cell proliferation and migration by upregulating Gli1 through an SMO-independent pathway, which may be beneficial to the treatment of CRC.

## Results

### Increased expression of Rab1A in CRC tissues

**Figure 1:**
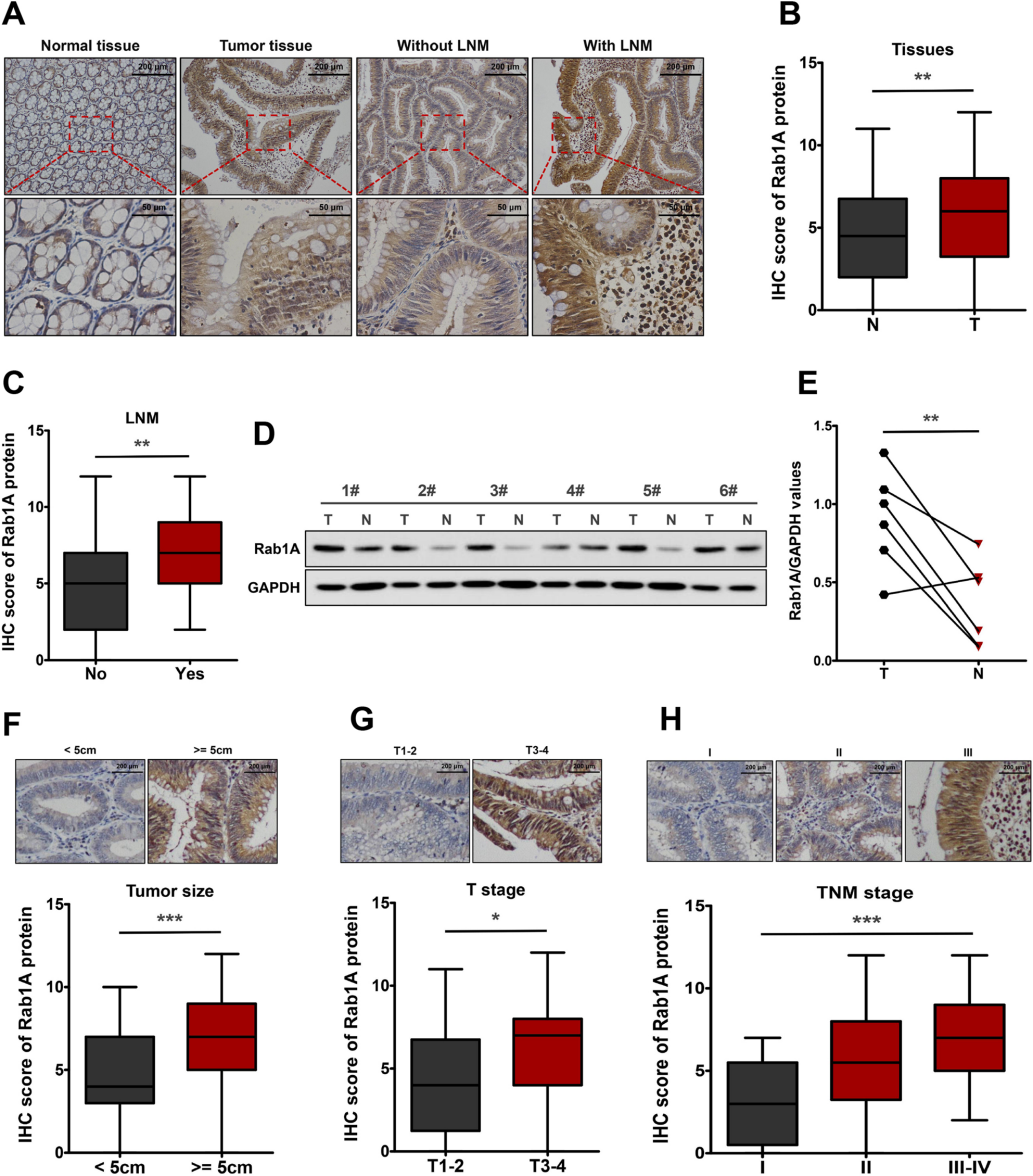
Rab1A is upregulated in CRC tissues. (**A**) IHC staining of Rab1A in CRC tumor tissues and the surrounding normal tissues. (**B**) IHC scores of Rab1A in CRC tumor tissues (T) and normal tissues (N). (**C**) IHC scores of Rab1A in CRC tumors with or without LNM. (**D**) Rab1A protein expression in six pairs of CRC tumors (T) and surrounding normal tissues (N) as detected by Western blotting. (**E**) Scatter plot analysis of Rab1A/GAPDH values in six paired CRC samples. (**F**) IHC staining and IHC scores of Rab1A in CRC tumors with different size. (**G**) IHC staining and IHC scores of Rab1A in CRC tumors with different T stage. H. IHC staining and IHC scores of Rab1A in CRC tumors with different TNM stage. Here cropped blots were displayed and all full-length blots are included in the Supplementary Information file. *P < 0.05, **P < 0.01, ***P < 0.001.

To explore whether Rab1A plays a role in CRC, we first set out to determine Rab1A expression in CRC tumor tissues and surrounding normal tissues. We performed IHC of collected CRC tissue samples and found that Rab1A expression markedly increased in tumor tissues in comparison to the normal tissues (*P* < 0.01, Fig. 1A,B). Further analysis showed that Rab1A expression level in human CRC tissues with lymph node metastasis (LNM) was higher than those without LNM (*P* < 0.01, Fig. 1A,C). We next performed Western blotting analysis to further explore Rab1A expression in CRC tissues. The results showed that the expression level of Rab1A in CRC tumor tissues was higher compared with that in the normal matched tissues (*P* < 0.01, Fig. 1D,E). Further IHC analysis revealed that compared to those with tumor size < 5 cm, Rab1A expression was higher in tumors ≥ 5 cm (*P* < 0.001, Fig. 1F). Furthermore, tumor tissues with deeper invasion (T3-4) exhibited higher Rab1A expression than those with T1-2 (*P* < 0.05, Fig. 1G). In addition, Rab1A expression levels were also positively correlated with the tumor-node-metastasis (TNM) stage of CRC patients (*P* < 0.001, Fig. 1H).

Next, the association between Rab1A expression and clinicopathological parameters in CRC was analyzed (Table 1). High Rab1A expression was markedly related to pathological large tumor size (χ^2^ = 12.289, *P* < 0.001), LNM (χ^2^ = 8.279, *P* = 0.004), and advanced TNM stage (χ^2^ = 17.467, *P* < 0.001). However, Rab1A expression showed no significant association with other clinicopathologic features, including age, gender, tumor location, degree of differentiation and depth of invasion (*P* > 0.05, Table 1).

**Table 1:** Relationship between Rab1A expression and clinicopathological characteristics of CRC patients.

Characteristics	Number of cases	Rab1A expression		χ^2^	*P*-value
		Low (n = 31)	High (n = 57)		
**Age (years)**					
< 65	33	8 (24.2%)	25 (75.8%)	2.792	0.095
≥ 65	55	23 (41.8%)	32 (58.2%)		
**Gender**					
Male	59	22 (37.3%)	37 (62.7%)	0.333	0.564
Female	29	9 (31.0%)	20 (69.0%)		
**Tumor location**					
Left-sided colon	26	12 (46.2%)	14 (53.8%)	2.334	0.311
Right-sided colon	30	8 (26.7%)	22 (73.3%)		
Rectum	32	11 (34.4%)	21 (65.6%)		
**Degree of differentiation**					
Well	75	27 (36.0%)	68 (64.0%)		1.000
Poor	13	4 (30.8%)	9 (69.2%)		
**Tumor size**					
< 5 cm	43	23 (53.5%)	20 (46.5%)	12.289	0.000***
> = 5 cm	45	8 (17.8%)	37 (82.2%)		
**Depth of invasion**					
T1-2	16	9 (56.3%)	7 (43.7%)	3.788	0.052
T3-4	72	22 (30.6%)	50 (69.4%)		
**Lymph node metastasis**					
Yes	50	24 (48.0%)	26 (52.0%)	8.279	0.004**
No	38	7 (18.4%)	31 (81.6%)		
**TNM stage**					
I	13	9 (69.2%)	4 (30.8%)	17.467	0.000***
II	36	14 (38.9%)	22 (61.1%)		
III–IV	39	8 (20.5%)	31 (79.5%)		

**P < 0.01, ***P < 0.001.

Collectively, these results suggested that Rab1A was upregulated in CRC and high Rab1A expression correlated with unfavorable clinicpathological characteristics of CRC patients.

### Rab1A promoted cell proliferation, migration and EMT progress in CRC

**Figure 2:**
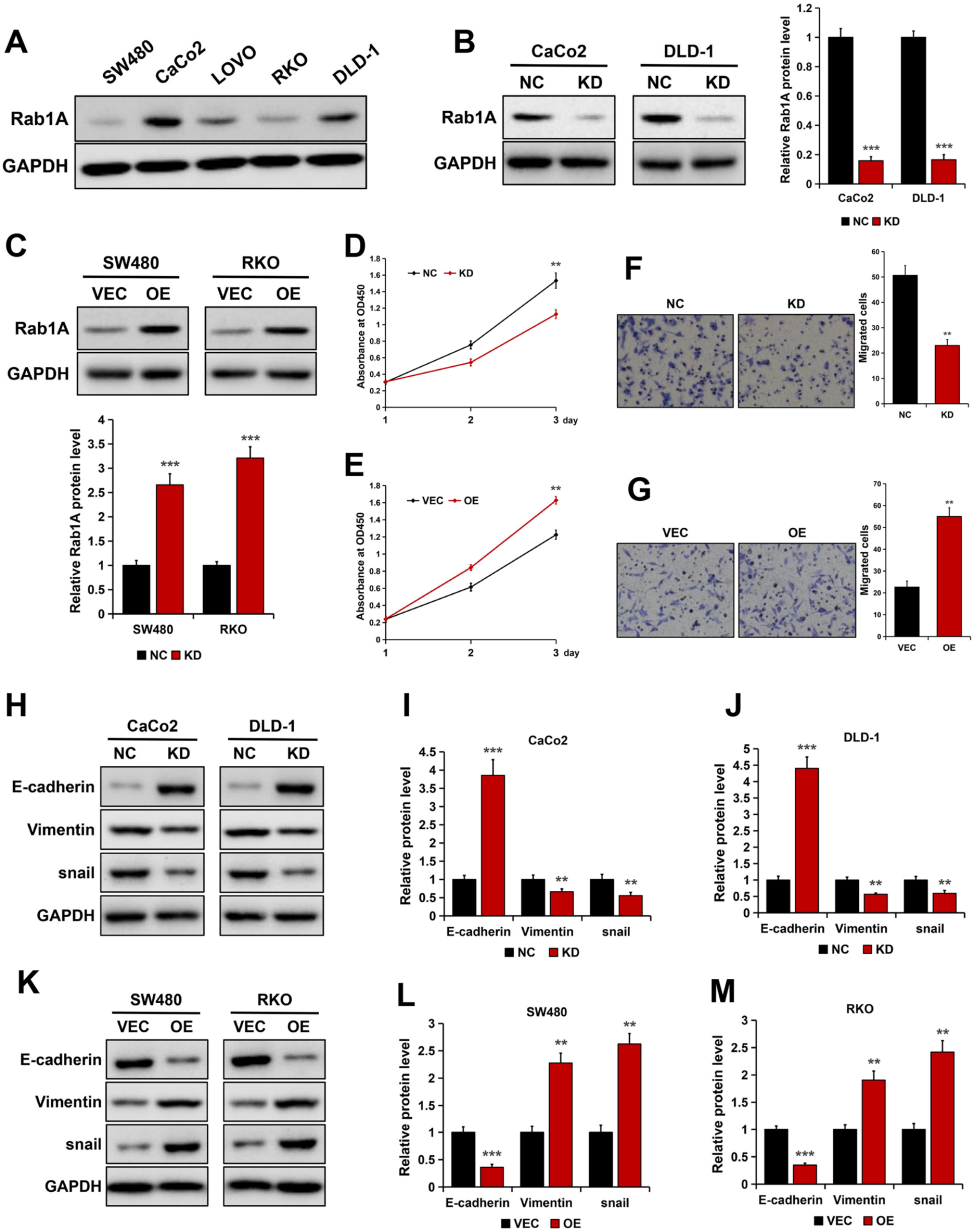
Rab1A promoted cell proliferation, migration and EMT in CRC cells. (**A**) Rab1A expression in five CRC cell lines as detected by Western blotting. (**B**) Rab1A expression was detected by Western blotting in CaCo2 and DLD-1 cells stably transfected with either control shRNA (NC) or Rab1A shRNA (KD). (**C**) Rab1A expression was detected by Western blotting in SW480 and RKO cells stably transfected with either empty vector (VEC) or plasmids encoding human Rab1A (OE). (**D**) CCK8 assays of Rab1A-silenced CaCo2 cells. (**E**) CCK8 assays of Rab1A-overexpressed SW480 cells. (**F**) Migration ability of Rab1A-silenced CaCo2 cells as detected by the Transwell assays. (**G**) Migration ability of Rab1A-overexpressed SW480 cells as detected by the Transwell assays. (**H**–**J**) E-cadherin, Vimentin and Snail expression in Rab1A-silenced cells as detected by Western blotting (**H**). The bands were quantified and presented as the mean ± S.E.M of three independent experiments (**I** and **J**). (**K**–**M**). E-cadherin, Vimentin and Snail expression in Rab1A-overexpressed cells as detected by Western blotting (**K**). The bands were quantified and presented as the mean ± S.E.M of three independent experiments (**L** and **M**). Here cropped blots were displayed and all full-length blots are included in the Supplementary Information file. **P < 0.01, ***P < 0.001.

To determine the biological functions of Rab1A in CRC progression, the expression levels of Rab1A in five CRC cell lines including SW480, CaCo2, LOVO, RKO and DLD-1 were detected using Western blotting. The results showed higher protein levels of Rab1A in CaCo2 and DLD-1 cells, compared to the low Rab1A protein expression in SW480 and RKO cell lines (Fig. 2A). Next, we stably knocked down Rab1A expression in CaCo2 and DLD-1 cells with relatively high endogenous Rab1A level using a lentivirus vector-based shRNA technique, and the knockdown efficiency was determined by Western blotting. The results showed that Rab1A protein level was markedly lower in CaCo2 and DLD-1 cells transfected with Rab1A-targeting shRNA than those transfected with control-shRNA (*P* < 0.001, Fig. 2B). We selected SW480 and RKO cells with relatively low endogenous Rab1A expression to construct stable Rab1A-overexpressed CRC cells. The results from Western blotting showed that Rab1A protein expression was greatly upregulated in cells stably transfected with plasmids encoding human Rab1A compared to those transfected with empty vector (P < 0.001, Fig. 2C).

To examine the potential oncogenic role of Rab1A in CRC cell proliferation, CCK8 assays were performed. The results showed that Rab1A depletion greatly impaired the proliferation ability of CRC cells (*P* < 0.01, Fig. 2D), whereas RablA did the opposite in SW480 cells (*P* < 0.01, Fig. 2E). To determine the effect of Rab1A on the migration of CRC cells, we conducted a Transwell migration test. The results showed that compared with the control group, the migration ability of Rab1A-depleted CaCo2 cells was significantly reduced (*P* < 0.01, Fig. 2F). However, Rab1A overexpression greatly enhanced the the migration ability of SW480 cells (*P* < 0.01, Fig. 2G).

To further explore the effect of Rab1A on CRC cell metastasis, we used Western blotting analysis to detect the expression of epithelial-mesenchymal transition (EMT)-related proteins (E-cadherin and Vimentin) and EMT-inducing transcription factor (such as Snail). The results showed that the protein expression of the epithelial marker E-cadherin was greatly increased in Rab1A-depleted CaCo2 and DLD-1 cells, while the expression of the mesenchymal marker Vimentin and the transcription factor Snail were significantly decreased (Fig. 2H–J). Similarly, the EMT protein markers and Snail were also examined in Rab1A-overexpressed SW480 and RKO cells. The results from Western blotting showed that Rab1A overexpression greatly inhibited E-cadherin expression, but promoted Vimentin and Snail expression (Fig. 2K–M).

### Rab1A upregulated Gli1 expression in an SMO-independent manner

**Figure 3:**
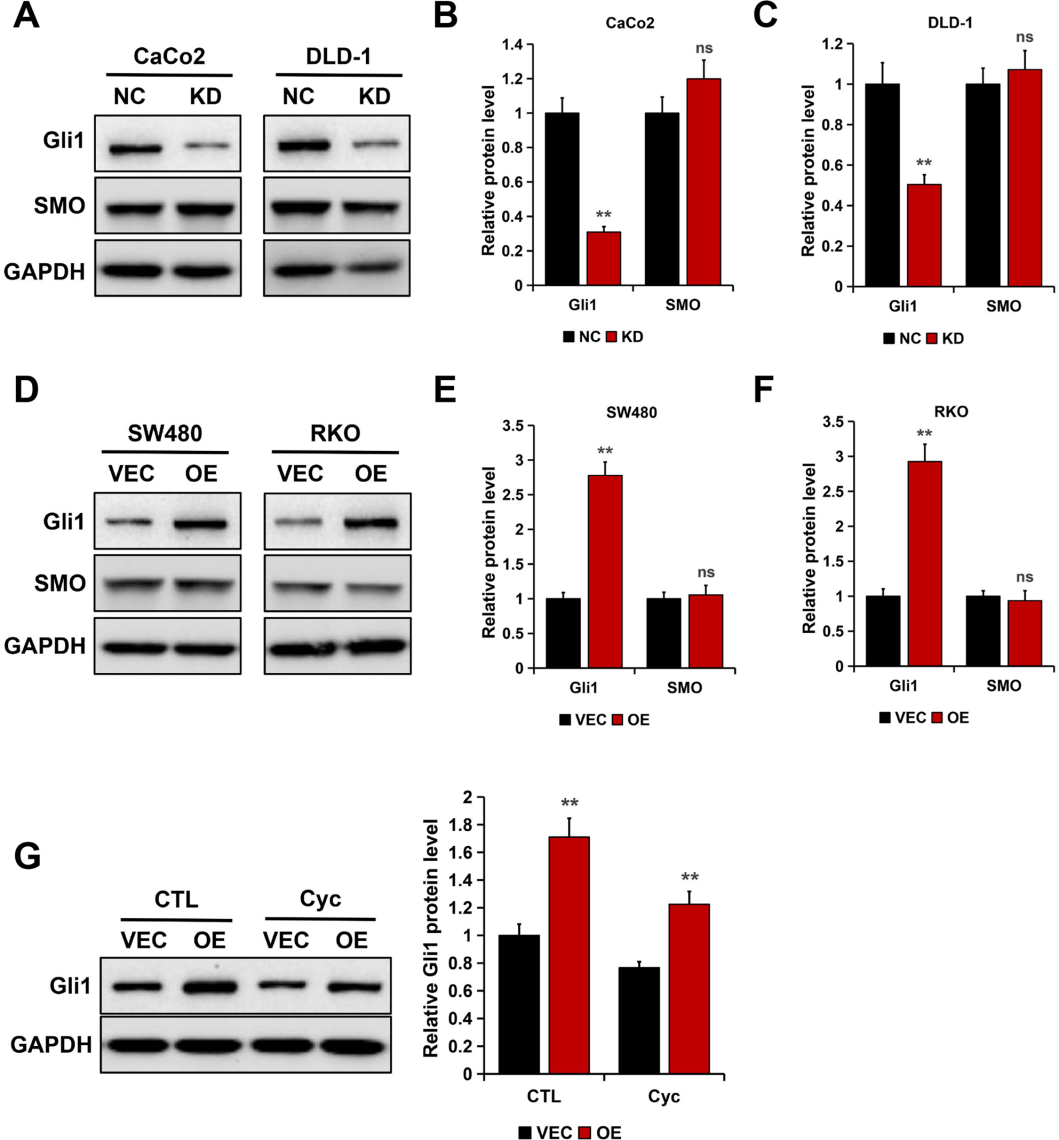
Rab1A upregulated Gli1 expression in an SMO-independent manner. (**A**–**C**). Gli1 and SMO expression in Rab1A-silenced cells as detected by Western blotting (**A**). The bands were quantified and presented as the mean ± S.E.M of three independent experiments (**B** and **C**). (**D**–**F**). Gli1 and SMO expression in Rab1A-overexpressed cells as detected by Western blotting (**D**). The bands were quantified and presented as the mean ± S.E.M of three independent experiments (**E** and **F**). (**G**) Gli1 expression is detected by Western blotting in Rab1A-overexpressed SW480 cells treated with cyclopamine (Cyc; 5 μM) for 24 h. Here cropped blots were displayed and all full-length blots are included in the Supplementary Information file. ns, nonsignificant, **P < 0.01.

Considering that Rab1A expression positively correlates with Gli1 in gastric cancer^27^ and aberrant Gli1 expression plays a vital role in the development of CRC^21,28^, we speculated whether Gli1 is regulated by Rab1A in CRC cells. The results from Western blotting showed that Gli1 protein levels were significantly decreased in Rab1A-depleted CaCo2 and DLD-1 cells (*P* < 0.01, Fig. 3A–C). On the contrary, the ectopic expression of Rab1A in SW480 and RKO cells resulted in increased levels of Gli1 (*P* < 0.01, Fig. 3D–F).

It is well known that SMO activates the Gli1 in the canonical Hedgehog pathway^20,24^. To investigate whether SMO is implicated in Rab1A-mediated Gli1 upregulation, we detected SMO protein expression in Rab1A-depleted and Rab1A-overexpressed CRC cells. The results from Western blotting showed that there was minimal effect of Rab1A on SMO protein expression in CRC cells (*P* > 0.05, Fig. 3). In addition, we treated Rab1A-overexpressed CRC cells with SMO inhibitor cyclopamine (Cyc). The results showed that cyclopamine did not affect the Rab1A-induced Gli1 upregulation (Fig. 3G), suggesting that Rab1A regulates Gli1 expression in an SMO-independent manner in CRC cells.

### Rab1A promoted cell proliferation and migration through a Gli1-dependent pathway

**Figure 4:**
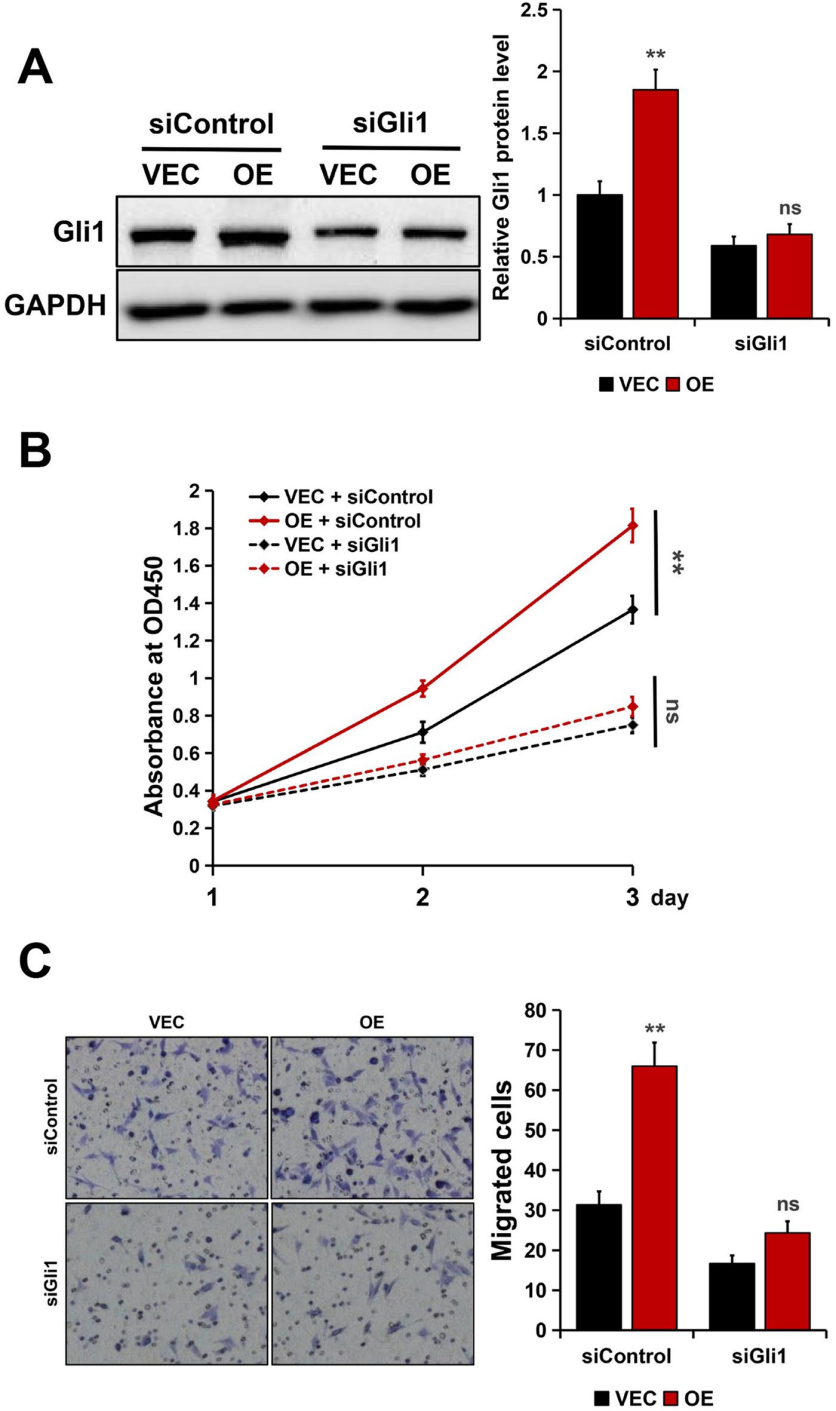
Rab1A promoted cell proliferation and migration through a Gli1-dependent pathway in SW480 cells. (**A**) Gli1 expression is detected by Western blotting in Rab1A-overexpressed SW480 cells treated control siRNA (siControl) or Gli1 siRNA (siGli1). (**B**) Cell proliferation ability was detected by CCK8 assays in Rab1A-overexpressed SW480 cells treated control siControl or siGli1. (**C**) Migration ability was detected by the Transwell assays in Rab1A-overexpressed SW480 cells treated control siControl or siGli1. Here cropped blots were displayed and all full-length blots are included in the Supplementary Information file. ns, nonsignificant, **P < 0.01, ***P < 0.001.

To assess the function of Gli1 in Rab1A-mediated enhancement of CRC cell proliferation and migration ability, Gli1 was silenced in Rab1A-overexpressed SW480 cells. The results from Western blotting showed that Gli1 protein expression was decreased in cells transfected with Gli1-specific siRNA as compared to the control cells (Fig. 4A). Furthermore, CCK8 assays showed that Rab1A overexpression promoted the proliferation of SW480 cells (*P* < 0.001, Fig. 4B), but the enhanced proliferation ability was greatly abolished when cells lacked Gli1 (*P* > 0.05, Fig. 4B). Next, Transwell migration assays were conducted to determine the effect of Rab1A on cell migration in the absence of Gli1. As shown in Fig. 4C, the migrated cell number was significantly increased in Rab1A-overexpressed SW480 cells (*P* < 0.01), whereas Rab1A overexpression failed to promote cell migration when cells lacked Gli1 (*P* > 0.05).

### Rab1A activated mTOR signaling in CRC cells

**Figure 5:**
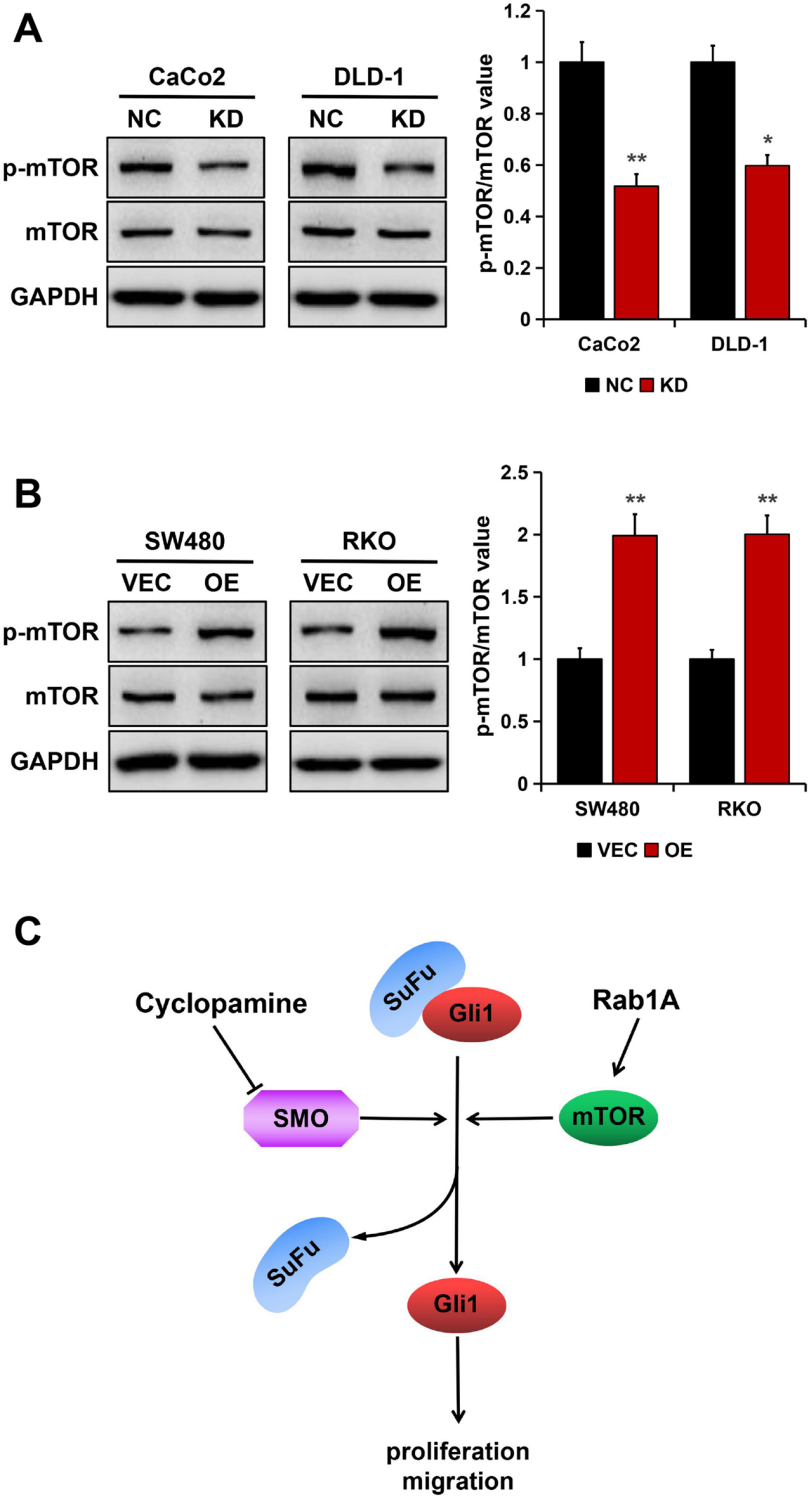
Rab1A regulated the phosphorylation of mTOR in CRC cells. (**A**) p-mTOR and mTOR expression in Rab1A-silenced cells as detected by Western blotting. (**B**) p-mTOR and mTOR expression in Rab1A-overexpressed cells as detected by Western blotting. (**C**) A proposed model for SMO-independent Gli1 upregulation regulated by Rab1A. Here cropped blots were displayed and all full-length blots are included in the Supplementary Information file. *P < 0.05, **P < 0.01.

It has been reported that Rab1A activates mTOR signaling in CRC cells^16,17^, and activated mTOR can upregulate Gli1 expression in an SMO-independent manner^24^. Therefore, we asked whether the mTOR signaling is involved in Rab1A-mediated Gli1 upregulation. The results from Western blotting showed that Rab1A depletion inhibited the phosphorylation of mTOR, with no effect on the total mTOR level (Fig. 5A). Conversely, ectopic Rab1A notably increased the phosphorylation of mTOR, but not the total mTOR level (Fig. 5B). Collectively, our results revealed that Rab1A activated mTOR signaling in CRC cells. Therefore, our results indicated that Rab1A promoted CRC cell proliferation and migration by upregulating Gli1 through an SMO-independent pathway (Fig. 5C).

## Discussion

In cancers, the role of Rab1A is contentious and it could be either oncogenic or tumor suppressive. Rab1A was described as an oncogene in gastric cancer^8^, nasopharyngeal carcinoma^11^, breast cancer^12^, lung cancer^13^ and hepatocellular carcinoma^14^. On the other hand, Rab1A expression was reduced and played anti-carcinogenic roles in androgen-independent prostate cancer^15^. In CRC, Rab1A was proposed as an mTORC1 activator and a colorectal oncogene^16,17^, but the concrete molecular mechanism of Rab1A in CRC is elusive and remains to be further explored..

In the present work, our data confirmed increased expression of Rab1A in human CRC tumor tissues in comparison to the normal tissues. We also found that Rab1A expression level was further increased in tumors with positive LNM, larger tumor sizes, deeper invasion and advanced TNM stage. On functional verification, our loss-of-function and gain-of-function experiments revealed that Rab1A knockdown or overexpression can respectively inhibit or promote the proliferation and migration of CRC cells.

EMT plays a crucial role in tumor cell metastasis, which is accompanied by upregulation of mesenchymal-associated markers (such as Vimentin) and downregulation of epithelial-associated markers (such as E-cadherin)^29^. In this study, we found for the first time that Rab1A promoted EMT progress in CRC cells evidenced by the facts that Rab1A knockdown increased E-cadherin expression and decreased Vimentin expression whereas Rab1A overexpression did the opposite, which is in accordance with the previous study in nasopharyngeal carcinoma^11^.

Gli1, as a key transcriptional factor of the HH pathway, has been proposed as an oncogene in CRC^21,28^. It was recently reported that Rab1A expression positively correlated with Gli1 in gastric cancer tissues^27^, but whether Gli1 is regulated by Rab1A in CRC cells is unknown. In the present study, we provided first evidence that Rab1A depletion suppressed Gli1 expression whereas ectopic Rab1A upregulated Gli1 expression in CRC cells. To detect whether Gli1 is implicated in Rab1A-mediated regulation of CRC cell proliferation and migration, Gli1 was silenced in Rab1A-overexpressed CRC cells. The results showed that Rab1A overexpression promoted the proliferation and migration of SW480 cells, but the enhanced proliferation and migration ability was greatly abolished when Gli1 was silenced, suggesting Rab1A-mediated regulation of CRC cell proliferation and migration is dependent on Gli1.

In mammals, the canonical HH signaling pathway initiates with binding of HH ligands to PTCH, causing the release of SMO, which subsequently activates the Gli1 transcription factors^21^. When SMO is inactivated, SuFu binds to and inhibits Gli1 function^24^. In this study, SMO inhibitor, such as cyclopamine, do not seem to affect Rab1A-mediated Gli1 upregulation, suggesting that Rab1A regulates Gli1 expression independently of SMO. Several SMO inhibitors have been tested in clinical trials^30^. However, in the present study, we revealed a SMO-independent regulation of Gli1 by Rab1A, which cannot be suppressed by SMO inhibitors. These results strongly suggest that combined inhibition of SMO and Rab1A may be a effective therapeutic target for the treatment of CRC. This also suggests that it is necessary and urgent to develop novel inhibitors of Rab1A, which warrants future investigation.

The regulation of mTOR signaling by Rab1A was reported by several studies in multiple types of cancers including CRC. In gastric cancer cells^8^, Rab1A knockdown was showed to repress proliferation and promote apoptosis by inhibiting mTOR/p70S6K signaling pathway. It was also reported that aberrant amino acid signaling promotes growth and metastasis of through Rab1A-dependent activation of mTORC1 in hepatocellular carcinomas^14^. In CRC^16,17^, Rab1A was reported as an mTORC1 activator and can promote the proliferation and migration ability via the mTOR/S6K1 pathway. In this study, we provided further evidences that Rab1A activated mTOR signaling in CRC cells. Considering activated mTOR can upregulate Gli1 expression independent of SMO^24^, we speculated that the mTOR signaling may be involved in Rab1A-mediated Gli1 upregulation, which needs further investigation in the future.

In summary, our data indicated that Rab1A expression was significantly upregulated in CRC tissues and increased Rab1A expression correlated with unfavorable clinicpathological features of CRC patients. We also found that Rab1A exerts its promotive effect on CRC cell proliferation, migration and EMT progress. Our study defines a mechanism for the function of Rab1A that activates mTOR/Gli1 signaling pathway, and then promotes malignant properties of CRC cells. Therefore, the Rab1A/mTOR/Gli1 axis may serve as a promising therapeutic target for the treatment of CRC.

## Materials and methods

### CRC tissues and cell lines

CRC tissue samples were obtained from patients underwent surgery in the Suzhou Wuzhong People’s Hospital from January 2010 to January 2018. Informed consent was obtained from all the patients. All procedures performed in this study involving human participants were in compliance with the Declaration of Helsinki. The present study was approved by the Ethics Committee of Wuzhong People's Hospital. The clinicopathological features of CRC patients are shown in Table 1.

The CRC cell lines were procured from Type Culture Collection of Chinese Academy of Sciences, Shanghai, China. Cells were cultured in RPMI 1640 (Gibco) medium supplemented with 10% foetal bovine serum (FBS; Gibco) and 1% penicillin/streptomycin (Invitrogen). All cells were cultured in a 5% CO_2_ incubation chamber at 37 °C. Some cells were treated with cyclopamine (Selleck).

### Immunohistochemistry (IHC)

All formalin-fixed, paraffin-embedded samples were cut into 4 μm thick sections. Briefly, Slides were immunoreacted overnight at 4 °C with the anti-Rab1A primary antibody (diluted at 1:100; 11671-1-AP; Proteintech) and then incubated with the secondary antibody for 1 h at room temperature. Hematoxylin was used for counterstaining. IHC score was independently assessed by two pathologists without knowledge of patient characteristics. For IHC score, the percentage (designated as 1 (< 25%), 2 (25–50%), 3 (51–75%) and 4 (> 75%)) of stained tumor cells was multiplied by the intensity (0, 1, 2, or 3) to achieve a total score between 0 and 12.

### Western blotting assay

Total proteins were extracted, subjected to SDS-PAGE gels and transferred to PVDF membranes. After blocking with 5% milk, the membrane was incubated with primary antibody overnight at 4 °C followed by incubation with secondary antibody. Signals were detected using chemiluminescence (Tanon, China). Primary antibodies used for immunoblotting were listed as followings: anti-E-cadherin antibody (diluted at 1:1000; 20874-1-AP; Proteintech), anti-Vimentin antibody (diluted at 1:1000; 10366-1-AP; Proteintech), anti-Gli1 antibody (diluted at 1:1000; 66905-1-Ig; Proteintech), anti-SMO antibody (diluted at 1:1000; 66851-1-Ig; Proteintech), anti-p-mTOR (diluted at 1:1000; 9205; CST), anti-mTOR (diluted at 1:1000; 2983; CST), anti-GAPDH (diluted at 1:1000; 60004-1-Ig; Proteintech).

### Construction of stable cell lines

CRC cells stably expressing Rab1A-specific short hairpin RNA (shRNA) and plasmids encoding human Rab1A were constructed to downregulate and upregulate Rab1A respectively using a lentivirus technique (GeneChem, Shanghai, China). Transfection of small interfering RNA (siRNA) targeting Gli1 was conducted using Lipofectamine™ RNAiMax (Invitrogen) at a final concentration of 20 nM according to the manufacturer’s instructions. The Rab1A shRNA target sequence is 5’-TCG TTG CAT TCT TAG CAC TGG-3’. The Gli1 siRNA target sequence is 5′-CUC CAC AGG CAU ACA GGAU-3′.

### Cell viability assay

Cell proliferation ability was determined using a Cell Counting Kit-8 (CCK-8) Assay (APExBIO) according to the manufacturer’s methods. Approximately 2000 cells were plated in 96-well plates for 1, 2 and 3 days. Then the cells were treated with 10 μl of CCK-8 solution and incubated for 2 h. Optical density (OD) was measured at 450 nm.

### Transwell migration assay

Cells resuspended in serum-free RPMI 1640 medium were seeded in the upper transwell chamber (Corning, USA). RPMI 1640 medium containing 10% FBS was added into the lower chamber. 4% paraformaldehyde (Beyotime, Beijing, China) was used to fix cells transferred to the lower surface. 0.1% crystal solution (Beyotime, Beijing, China) was used to stain cells.

### Statistical analysis

Data of all assays were expressed as means ± standard errors of the mean (SEM). A two-tailed Student’s *t*-test was used for analysing the statistical difference between two data sets while the multi-set data groups were analysed through one way analysis of variance (ANOVA). Analysis of IHC was evaluated using the chi-square statistical test or Fisher’s exact test. *P* < 0.05 was considered statistically significant.

## Supplementary Information

Supplementary Information.

## References

[1] Jacobs D, Zhu R, Luo J, Grisotti G, Heller D, Kurbatov V, Johnson CH, Zhang Y, Khan SA (2018). Defining early-onset colon and rectal cancer. Front. Oncol..

[2] Tfaily MA, Naamani D, Kassir A, Sleiman S, Ouattara M, Moacdieh MP, Jaffa MA (2019). Awareness of colorectal cancer and attitudes towards its screening guidelines in Lebanon. Ann. Global Health.

[3] Yu IS, Cheung WY (2018). Metastatic colorectal cancer in the era of personalized medicine: A more tailored approach to systemic therapy. Can. J. Gastroenterol. Hepatol..

[4] Carethers JM, Jung BH (2015). Genetics and genetic biomarkers in sporadic colorectal cancer. Gastroenterology.

[5] Siena S, Sartore-Bianchi A, Di Nicolantonio F, Balfour J, Bardelli A (2009). Biomarkers predicting clinical outcome of epidermal growth factor receptor-targeted therapy in metastatic colorectal cancer. J. Natl. Cancer Inst..

[6] Yan T, Shen C, Jiang P, Yu C, Guo F, Tian X, Zhu X, Lu S, Han B, Zhong M, Chen J, Liu Q, Chen Y, Zhang J, Hong J, Chen H, Fang JY (2021). Risk SNP-induced lncRNA-SLCC1 drives colorectal cancer through activating glycolysis signaling. Signal Transduct Target Ther..

[7] Hutagalung AH, Novick PJ (2011). Role of Rab GTPases in membrane traffic and cell physiology. Physiol. Rev..

[8] Li Z, Li Y, Jia Y, Ding B, Yu J (2020). Rab1A knockdown represses proliferation and promotes apoptosis in gastric cancer cells by inhibition of mTOR/p70S6K pathway. Arch. Biochem. Biophys..

[9] Charng WL, Yamamoto S, Jaiswal M, Bayat V, Xiong B, Zhang K, Sandoval H, David G, Gibbs S, Lu HC, Chen K, Giagtzoglou N, Bellen HJ (2014). Drosophila Tempura, a novel protein prenyltransferase α subunit, regulates notch signaling via Rab1 and Rab11. PLoS Biol..

[10] Wang C, Yoo Y, Fan H, Kim E, Guan KL, Guan JL (2010). Regulation of Integrin β1 recycling to lipid rafts by Rab1a to promote cell migration. J. Biol. Chem..

[11] Yang XZ, Chen XM, Zeng LS, Deng J, Ma L, Jin C, Wang R, Wang MH, Wen YF, Wu XL, Wang HY, Cui SZ (2020). Rab1A promotes cancer metastasis and radioresistance through activating GSK-3β/Wnt/β-catenin signaling in nasopharyngeal carcinoma. Aging (Albany NY).

[12] Xu H, Qian M, Zhao B, Wu C, Maskey N, Song H, Li D, Song J, Hua K, Fang L (2017). Inhibition of RAB1A suppresses epithelial-mesenchymal transition and proliferation of triple-negative breast cancer cells. Oncol. Rep..

[13] Wang X, Liu F, Qin X, Huang T, Huang B, Zhang Y, Jiang B (2016). Expression of Rab1A is upregulated in human lung cancer and associated with tumor size and T stage. Aging (Albany NY).

[14] Xu BH, Li XX, Yang Y, Zhang MY, Rao HL, Wang HY, Zheng XF (2015). Aberrant amino acid signaling promotes growth and metastasis of hepatocellular carcinomas through Rab1A-dependent activation of mTORC1 by Rab1A. Oncotarget.

[15] Sun T, Wang X, He HH, Sweeney CJ, Liu SX, Brown M, Balk S, Lee GS, Kantoff PW (2014). MiR-221 promotes the development of androgen independence in prostate cancer cells via downregulation of HECTD2 and RAB1A. Oncogene.

[16] Thomas JD, Zhang YJ, Wei YH, Cho JH, Morris LE, Wang HY, Zheng XF (2014). Rab1A is an mTORC1 activator and a colorectal oncogene. Cancer Cell.

[17] Cheng Z, Shao X, Xu M, Wang J, Kuai X, Zhang L, Wu J, Zhou C, Mao J (2019). Rab1A promotes proliferation and migration abilities via regulation of the HER2/AKT-independent mTOR/S6K1 pathway in colorectal cancer. Oncol. Rep..

[18] Kong JH, Siebold C, Rohatgi R (2019). Biochemical mechanisms of vertebrate hedgehog signaling. Development.

[19] Chapouly C, Guimbal S, Hollier PL, Renault MA (2019). Role of hedgehog signaling in vasculature development, differentiation, and maintenance. Int. J. Mol. Sci..

[20] Jeng KS, Sheen IS, Leu CM, Tseng PH, Chang CF (2020). The role of smoothened in cancer. Int. J. Mol. Sci..

[21] Wu C, Zhu X, Liu W, Ruan T, Tao K (2017). Hedgehog signaling pathway in colorectal cancer: Function, mechanism, and therapy. Onco Targets Ther..

[22] Geyer N, Gerling M (2021). Hedgehog signaling in colorectal cancer: All in the stroma?. Int. J. Mol. Sci..

[23] Pietrobono S, Gagliardi S, Stecca B (2019). Non-canonical hedgehog signaling pathway in cancer: Activation of GLI transcription factors beyond smoothened. Front. Genet..

[24] Wang Y, Ding Q, Yen CJ, Xia W, Izzo JG, Lang JY, Li CW, Hsu JL, Miller SA, Wang X, Lee DF, Hsu JM, Huo L, Labaff AM, Liu D, Huang TH, Lai CC, Tsai FJ, Chang WC, Chen CH, Wu TT, Buttar NS, Wang KK, Wu Y, Wang H, Ajani J, Hung MC (2012). The crosstalk of mTOR/S6K1 and Hedgehog pathways. Cancer Cell.

[25] Teperino R, Aberger F, Esterbauer H, Riobo N, Pospisilik JA (2014). Canonical and non-canonical Hedgehog signalling and the control of metabolism. Semin. Cell Dev. Biol..

[26] Xu M, Wang J, Li H, Zhang Z, Cheng Z (2020). AIM2 inhibits colorectal cancer cell proliferation and migration through suppression of Gli1. Aging (Albany NY)..

[27] Shao X, Cheng Z, Xu M, Mao J, Wang J, Zhou C (2019). Prognosis, significance and positive correlation of Rab1A and p-S6K/Gli1 expression in gastric cancer. Anticancer Agents Med. Chem..

[28] Yang Z, Zhang C, Qi W, Cui Y, Xuan Y (2018). GLI1 promotes cancer stemness through intracellular signaling pathway PI3K/Akt/NFκB in colorectal adenocarcinoma. Exp. Cell Res..

[29] Gonzalez DM, Medici D (2014). Signaling mechanisms of the epithelial-mesenchymal transition. Sci. Signal.

[30] Quaglio D, Infante P, Di Marcotullio L, Botta B, Mori M (2020). Hedgehog signaling pathway inhibitors: An updated patent review (2015-present). Exp. Opin. Ther. Pat..

