# Supplementary material for: Rab1A promotes cell proliferation and migration by upregulating Gli1 in colorectal cancer
Source: Sci Rep. 2021 Aug 10;11:16243. doi: 10.1038/s41598-021-95798-7 (PMC8355269; doi:10.1038/s41598-021-95798-7)

Rab1A promotes cell proliferation and migration by upregulating Gli1 in colorectal cancer

Chaozhong Peng[1], Xiao Li[1], Zhixue Ye[1], Wenqing Wu[1, *]

[1]Department of General Surgery, Suzhou Wuzhong People's Hospital, Suzhou 215128, Jiangsu, China.

[*]Correspondence to: Dr. Wenqing Wu, Department of General Surgery, Suzhou Wuzhong People's Hospital, No. 61 Dongwu North Road, Suzhou 215128, Jiangsu, China. E-mail addresses: wuwenqing2021@163.com

Supplementary Figure S1. Western blot analysis of full-length gels and blots of Figure 1.

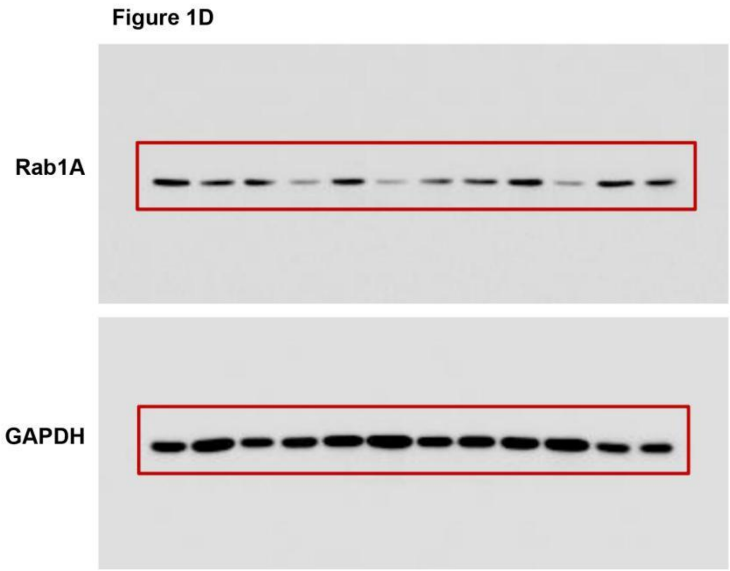

Supplementary Figure S2. Western blot analysis of full-length gels and blots of Figure 2.

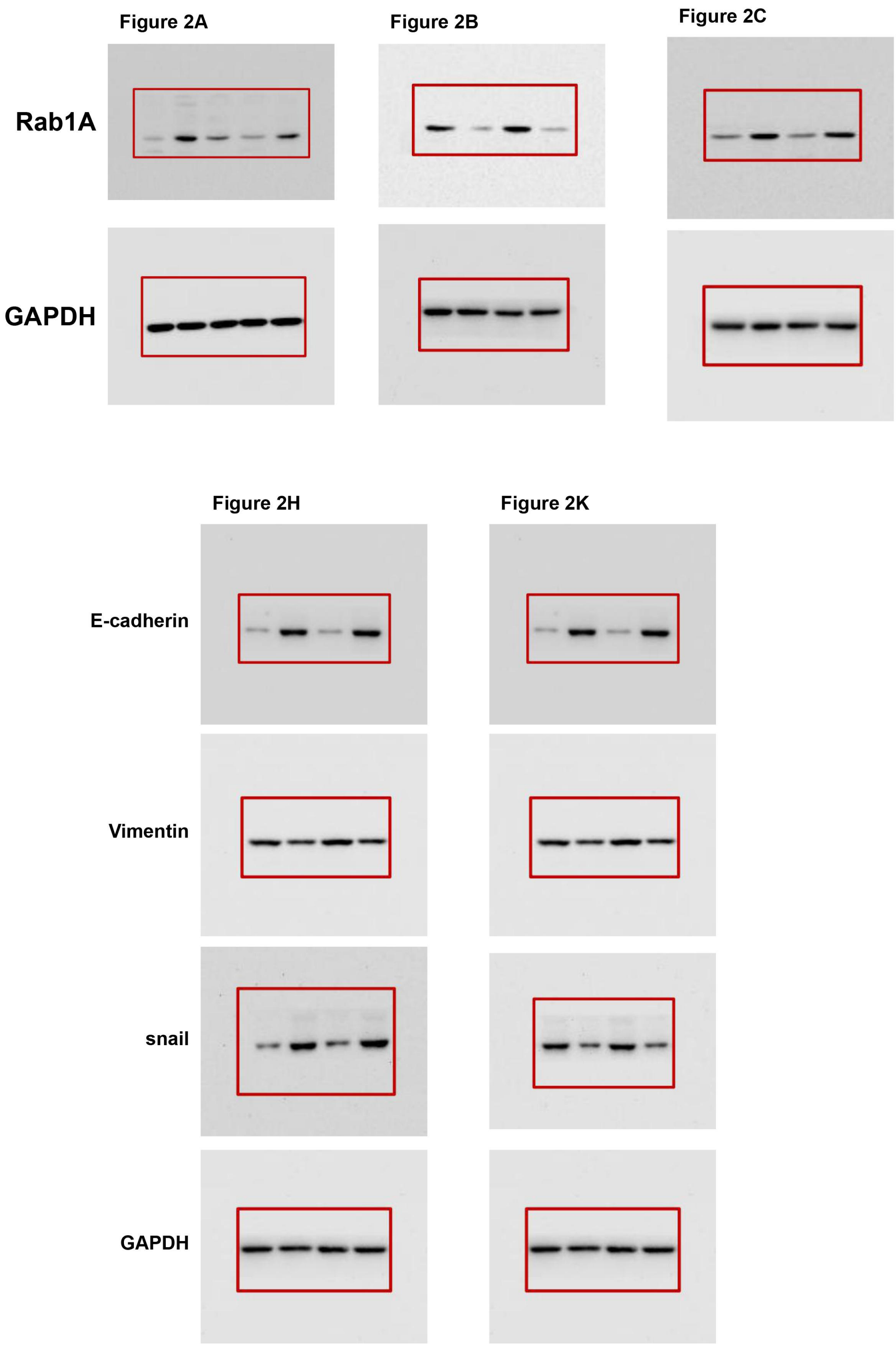

Supplementary Figure S3. Western blot analysis of full-length gels and blots of Figure 3.

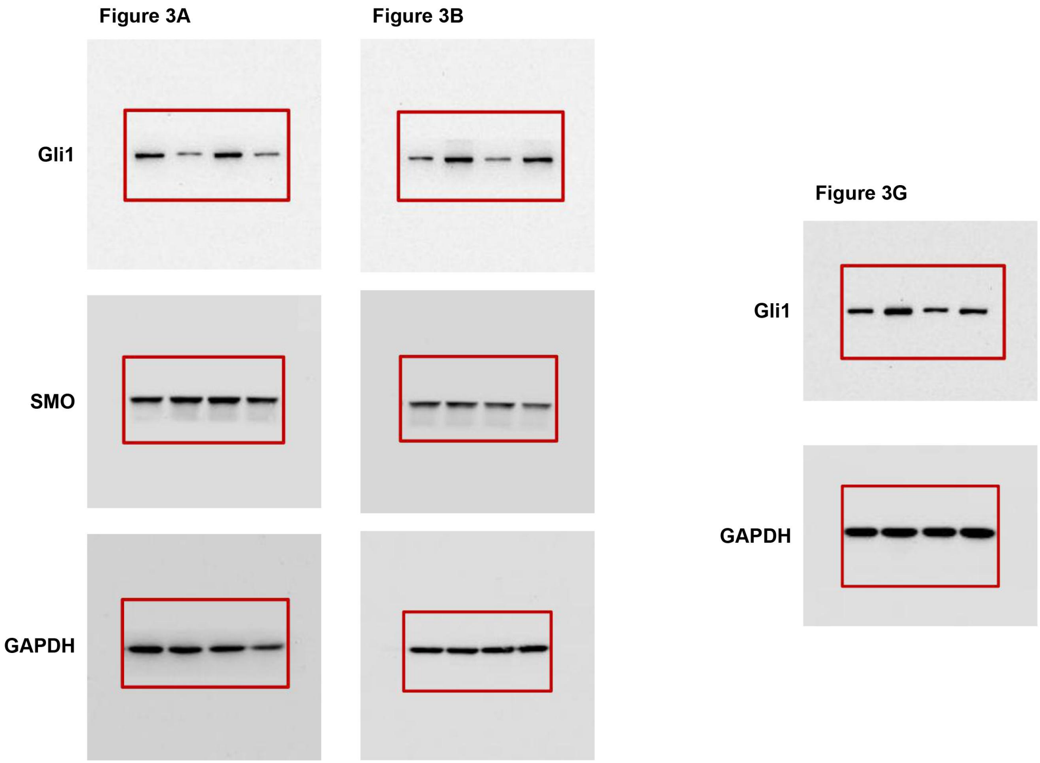

Supplementary Figure S4. Western blot analysis of full-length gels and blots of Figure 4.

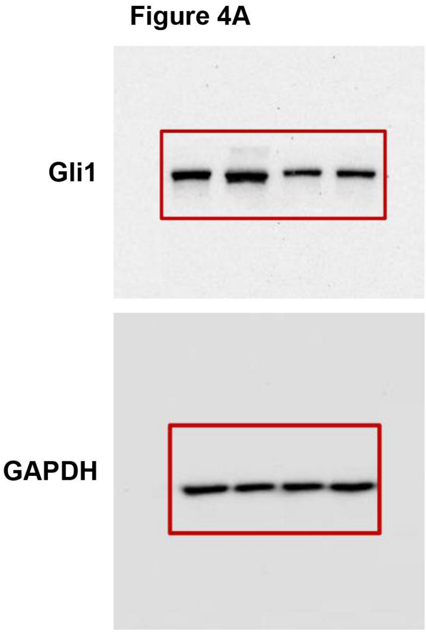

Supplementary Figure S5. Western blot analysis of full-length gels and blots of Figure 5.

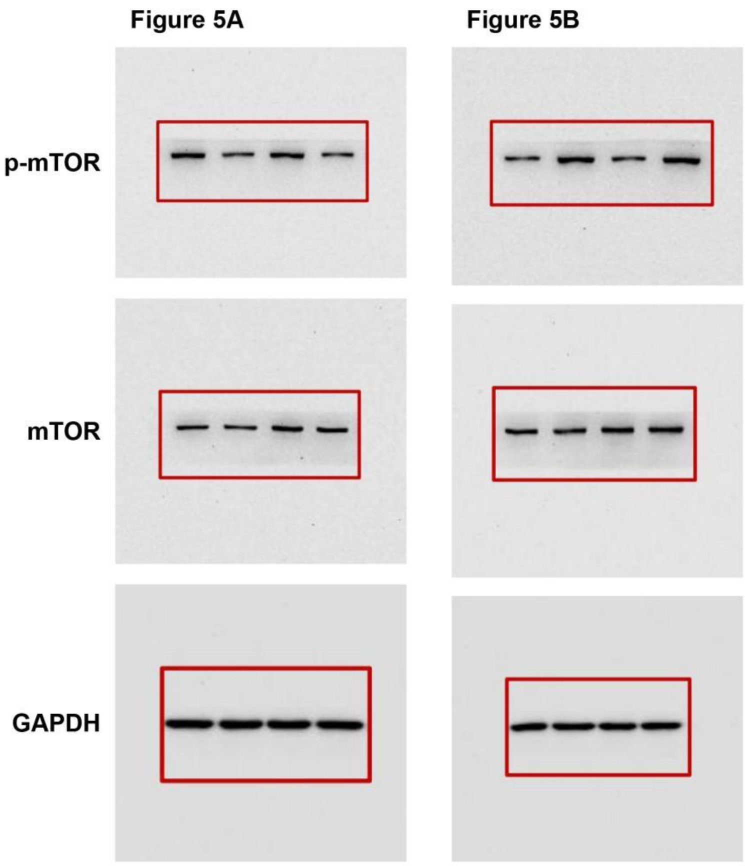

Supplement: Supplementary file 1 — Supplementary Information. [file 41598_2021_95798_MOESM1_ESM.pdf]
